# Oxytocin in the medial prefrontal cortex regulates maternal care, maternal aggression and anxiety during the postpartum period

**DOI:** 10.3389/fnbeh.2014.00258

**Published:** 2014-08-06

**Authors:** Sara Sabihi, Shirley M. Dong, Nicole E. Durosko, Benedetta Leuner

**Affiliations:** ^1^Department of Psychology, The Ohio State UniversityColumbus, OH, USA; ^2^Department of Neuroscience, The Ohio State UniversityColumbus, OH, USA

**Keywords:** medial prefrontal cortex, prelimbic, maternal behavior, lactation, anxiety, oxytocin

## Abstract

The neuropeptide oxytocin (OT) acts on a widespread network of brain regions to regulate numerous behavioral adaptations during the postpartum period including maternal care, maternal aggression, and anxiety. In the present study, we examined whether this network also includes the medial prefrontal cortex (mPFC). We found that bilateral infusion of a highly specific oxytocin receptor antagonist (OTR-A) into the prelimbic (PL) region of the mPFC increased anxiety-like behavior in postpartum, but not virgin, females. In addition, OTR blockade in the postpartum mPFC impaired maternal care behaviors and enhanced maternal aggression. Overall, these results suggest that OT in the mPFC modulates maternal care and aggression, as well as anxiety-like behavior, during the postpartum period. Although the relationship among these behaviors is complicated and further investigation is required to refine our understanding of OT actions in the maternal mPFC, these data nonetheless provide new insights into neural circuitry of OT-mediated postpartum behaviors.

## Introduction

The postpartum period is accompanied by dramatic behavioral changes in all mammalian species. In rats, females that were previously unresponsive or infanticidal towards pups will engage in an elaborate repertoire of caregiving activities after parturition that includes retrieval of displaced pups to the nest, nursing and/or crouching over pups, pup licking and grooming, as well as heightened aggression towards conspecifics (Rosenblatt, 1967; Erskine et al., 1978; Numan and Woodside, 2010). Together these behaviors serve to nurture and protect the young thus promoting their development and survival. In addition to offspring-directed behaviors and their defense against a potential threat, postpartum females also show changes in their emotional state characterized by attenuated levels of anxiety-like behavior (Fleming and Luebke, 1981; Hard and Hansen, 1985; Neumann et al., 2000a; Lonstein, 2005a, 2007; Figueira et al., 2008; Macbeth and Luine, 2010; Jurek et al., 2012). The co-occurrence of reduced anxiety along with heightened maternal responsiveness and aggression during the postpartum period suggests a link between emotionality and proper parenting (Fleming and Luebke, 1981; Lonstein, 2007).

Each of the behavioral changes that emerge postpartum is mediated by a vast array of neurochemicals, including oxytocin (OT). OT is a neurohormone synthesized in the supraoptic (SON) and paraventricular (PVN) nuclei of the hypothalamus. In postpartum rats, suckling simultaneously stimulates the release of OT from the pituitary into the bloodstream as well as into the CNS (Landgraf et al., 1992; Neumann et al., 1993; Landgraf and Neumann, 2004; Bosch and Neumann, 2012). In the periphery, OT enhances smooth muscle contractility for milk ejection while the effects of OT on maternal care, maternal aggression, and postpartum anxiety are mediated by CNS OT. However, both the central and peripheral actions of OT are transduced by a single isoform of the oxytocin receptor (OTR; Gimpl and Fahrenholz, 2001). During the peripartum period, expression of the OTR increases not only on mammary contractile cells but also within various regions of the brain including the lateral septum, medial preoptic area (MPOA), central nucleus of the amygdala (CEA), ventromedial nucleus of the hypothalamus, nucleus accumbens, olfactory bulb (OB), PVN, bed nucleus of the stria terminalis (BNST), and ventral tegmental area (VTA; Insel, 1990; Pedersen et al., 1994; Francis et al., 2000; Bosch et al., 2010; Caughey et al., 2011; Bosch and Neumann, 2012). Not surprisingly, many of these brain regions have been implicated as sites mediating the behavioral effects of OT during the postpartum period. For example, key areas involved in OT-induced maternal behavior include the MPOA (Pedersen et al., 1994; Bosch and Neumann, 2012), VTA (Pedersen et al., 1994; Shahrokh et al., 2010), and OB (Yu et al., 1996) while OT has been shown to influence maternal aggression through its effects in the PVN (Giovenardi et al., 1998; Bosch et al., 2004), BNST (Consiglio et al., 2005), and CEA (Lubin et al., 2003; Bosch et al., 2005; Consiglio et al., 2005). Although less studied, the postpartum-associated reduction in anxiety has been attributed to OT acting within the midbrain periaqueductal gray (PAG; Figueira et al., 2008), PVN (Jurek et al., 2012), and CEA (Bosch et al., 2005). Thus, OT acts on a widespread network of brain regions to influence postpartum behaviors.

Several lines of evidence suggest that another component of this network may include the medial prefrontal cortex (mPFC). First, in addition to expressing OTR (Insel and Shapiro, 1992; Gould and Zingg, 2003; Liu et al., 2005; Smeltzer et al., 2006), the mPFC contains OT-sensitive neurons (Ninan, 2011) and receives long-range axonal projections from OT producing neurons in the hypothalamus (Sofroniew, 1983; Knobloch et al., 2012). Second, the mPFC of postpartum rats becomes activated by suckling or OT administration (Febo et al., 2005; Febo, 2012) as well as during the display of maternal aggression (Gammie et al., 2004; Nephew et al., 2009). Third, lesion and inactivation studies have implicated the mPFC in the regulation of anxiety (Shah and Treit, 2003; Stern et al., 2010) and some aspects of maternal care (Afonso et al., 2007; Febo et al., 2010). Lastly, OT regulates social (Young et al., 2014) and anxiety (Sabihi et al., 2014) behaviors in female rodents at least in part through its actions in the prelimbic (PL) region of the mPFC. Although these findings collectively suggest that the mPFC may be a common target underlying the behavioral effects of OT during the postpartum period, this possibility has not been previously explored. Thus, in the present study, we examined maternal behavior, maternal aggression, and anxiety-like behavior in postpartum rats following administration of a highly specific OTR-A into the PL mPFC.

## Materials and methods

### Animals

Age matched adult (9–12 weeks of age) virgin (225–250 g) and timed pregnant [gestation day (GD) 14] female Sprague-Dawley rats from Taconic (Germantown, NY) were used. All rats were housed individually in a temperature and humidity controlled room and maintained on a 12/12 light/dark cycle (lights on at 06:00 h) with access to food and water ad libitum. All procedures were conducted in accordance with The Guide for the Care and Use of Laboratory Animals published by the National Institutes of Health and approved by The Ohio State University Institutional Animal Care and Use Committee.

For postpartum females, the day of birth was designated as postpartum day 0 (PD0) and on PD1 each litter was culled to five male and five female pups. In virgin females, stages of estrous were monitored through daily vaginal swabs which were taken at least 2 h prior to testing. Samples of cells were obtained with a sterile cotton swab saturated in 0.9% saline and applied to a glass slide. After drying, slides were stained with 1% aqueous Toluidine Blue and cell types characterized under 10X magnification (Everett, 1989). Only those virgin females that had normal 4–5 d estrous cycles were used.

### Surgical procedures

On GD16-17, rats were anesthetized with a 2–4% isoflurane gas/air mixture and aligned on a stereotaxic apparatus (Kopf Instruments, Tujunga, CA). This timepoint for surgery is consistent with prior studies assessing behavioral changes during the postpartum period following drug administration via cannulation (Neumann et al., 2000a; Lubin et al., 2003; Figueira et al., 2008). Body temperature was maintained throughout the surgery with a warming pad. Bilateral cannula guides (pedestal mounted 22-gauge stainless steel tubes with 1.5 mm separation and cut 3.5 mm below the pedestal; Plastics One, Roanoke, VA) were secured in a stereotaxic holder and lowered into the PL mPFC (AP: + 3.2 mm, ML: ± 0.75 mm, DV: −3.2 mm; Paxinos and Watson, 1998). The PL mPFC was targeted because it has been most consistently linked to maternal care, maternal aggression, and anxiety (Nephew et al., 2009; Febo et al., 2010; Pereira and Morrell, 2011; Febo, 2012; Sabihi et al., 2014). The cannula were secured by stainless steel screws and dental cement. A bilateral stainless steel obturator (0.35 mm diameter; Plastics One) extending 0.2 mm beyond the tip of the guide cannula was placed into the guide cannula after surgeries. The scalp was closed around the protruding portion of the cannula with sutures. Following surgery, rats were allowed to recover for at least 7 d before behavioral testing.

### Central infusions

On days 2 and 4 post-surgery, all rats were habituated to the handling and infusion procedures. During habituation, rats were removed from their home cage and handled for approximately 3 min while being lightly restrained in a terrycloth towel. The obturators were then removed and a 28-gauge bilateral injection cannula extending 0.2 mm beyond the tip of the guide cannula into the PL mPFC was inserted into the guide. The injection cannula were left in place for 3 min then removed and the obturator replaced. On testing days (during diestrus for virgin females and on PD3, PD5, and PD7 for postpartum rats), rats underwent the same procedure as described above except that an injection cannula attached to two 1 μl Hamilton Syringes via PE-10 tubing was inserted into the guide cannula. Bilateral infusions were made using a Harvard Apparatus Pico Plus Elite infusion pump (Holliston, MA) which delivered a 1.0 μl volume into each hemisphere over 3 min. The injector was left in place for an additional 1 min before withdrawal.

### Experimental design

To investigate the effects of OTR blockade in the mPFC, virgin and postpartum female rats received bilateral infusions of the highly specific OTR-A (Manning et al., 2012), desGly-NH_2_-d(CH_2_)_5_[D-Tyr^2^,Thr^4^]OVT (courtesy of Dr. Maurice Manning, University of Toledo) into the PL mPFC at a dose of either 0.1 μg/1 μl (*n* = 7 postpartum; *n* = 7 virgin) or 0.5 μg/1 μl (*n* = 8 postpartum; *n* = 8 virgin) into each hemisphere (Lubin et al., 2003; Bosch et al., 2005; Figueira et al., 2008). Additional groups of control rats received a 1 μl infusion of physiological saline (*n* = 8 postpartum; *n* = 8 virgin). Maternal behavior was assessed on PD3, when postpartum females exhibit high levels of maternal care (Afonso et al., 2007; Numan et al., 2009). Anxiety-like behavior was assessed on PD5, when postpartum females exhibit low levels of anxiety-like behavior (Lonstein, 2005a; Figueira et al., 2008). Maternal aggression was assessed on PD7, when aggressive behavior peaks (Caughey et al., 2011). Each postpartum female was evaluated on all behavioral tests, the timing of which corresponds to postpartum time points that have been used in prior studies (Lonstein, 2005a; Numan et al., 2009; Caughey et al., 2011). Only anxiety-like behavior was assessed in virgin females and this was done during diestrus in order to control for fluctuations in anxiety across the estrous cycle (Mora et al., 1996; Marcondes et al., 2001; Walf and Frye, 2007). Studies which have examined factors regulating anxiety-like behavior in virgin females commonly test during diestrus since this is the stage when anxiety is relatively stable (De Almeida et al., 1998; Marcondes et al., 2001; Figueira et al., 2008). In all cases, behavioral testing was done between 09:00–12:00 h 20 min after infusions (Ring et al., 2006; Nyuyki et al., 2011).

### Maternal behavior

Postpartum females (PD3) were brought to the infusion room in their home cage. Following a 20 min habituation period, their litters were removed and placed in a separate cage on a heating pad. Postpartum females were then were infused with either OTR-A or saline and returned to their home cage which was then placed in an adjacent testing room. 20 min after infusion, the mother’s own pups were reintroduced into the home cage in a scattered manner in the corner diagonally opposite to the nest. A 30 min video recording began immediately following the return of the pups. During maternal observations, the following latencies and/or durations (when appropriate) of behaviors were recorded: pup retrieval (pulling stray pups by the scruff back to the nest), pup directed behaviors which included all contact with pups except retrieval (sniffing, anogenital or body licking of pups, crouching over pups, nursing), exploratory behavior (exploration of the cage), rearing (standing on hind legs), and self-grooming. Animals that showed incomplete retrieval (not retrieving all 10 pups) were given a maximal retrieval latency of 1800 s.

### Anxiety-like behavior

Both postpartum females (PD5) and virgin females (diestrus) were tested for anxiety-like behavior using two well validated models—the EPM and the OF tests (Prut and Belzung, 2003; Lapiz-Bluhm et al., 2008; Rotzinger et al., 2010). Virgin females were included in this experiment in order to examine whether the OTR-A would prevent the reduction in anxiety typically observed postpartum as well as to confirm prior reports showing that the behavioral effects of OTR blockade are specific to the postpartum period (Neumann et al., 2000a; Figueira et al., 2008; Sabihi et al., 2014).

All females were brought into the infusion room in their home cage. Following a 20 min habituation period, litters were removed from postpartum females and placed in a separate cage on a heating pad. Virgin and postpartum females were then infused with either OTR-A or saline and returned to their home cage which was placed in an adjacent testing room. 20 min after infusion, anxiety testing began. The two anxiety tests were done 5 min apart on the same day and the order of these tests was counterbalanced among rats.

The EPM consisted of a cross-shaped platform (height: 50 cm) with four arms (width: 10 cm; length: 50 cm), two of which were enclosed by walls 50 cm in height. Rats were placed in the center of the platform (10 × 10 cm), facing a junction between an open and closed arm and allowed to explore for 5 min under bright light conditions (550 lux open arms, 150 lux closed arms). The number of entries into the open arms and the percentage of time spent in the open arms (time in open arms/time in open and closed arms × 100) were used as measures of anxiety-like behavior (Pellow et al., 1985; Cruz et al., 1994; Lapiz-Bluhm et al., 2008). An increase in the percentage of time spent in the open arms and a greater number of open arm entries are indicative of reduced anxiety. Locomotor activity was assessed using the number of closed arm entries (Pellow et al., 1985; Cruz et al., 1994; Lapiz-Bluhm et al., 2008).

For the OF test, a 60 × 60 cm Plexiglas arena with walls 40 cm high was used. The floor of the arena was covered with gridlines which allowed for measurement of locomotion. The gridlines were spaced 10 cm apart yielding a total of 36, 10 × 10 cm squares. The inner area was considered the central 16 squares which covered a 40 × 40 cm area. Rats were placed in the center of the open field and during a 5 min test, the percentage of time spent in the center of the arena (time spent in center/total time × 100) as well as the percentage of gridlines crossed in the center of the arena (number of center gridlines crossed/total number of gridlines crossed × 100) were used as measures of anxiety-like behavior. An increase in either measure correlates with lower anxiety. Locomotor activity was assessed using the total number of gridlines crossed (Prut and Belzung, 2003). Testing occurred in bright light conditions (550 lux inner zone, 150 lux outer zone).

### Maternal aggression

Aggressive behavior was assessed in postpartum females (PD7) using the maternal defense test (Neumann et al., 2001; Lubin et al., 2003; Bosch et al., 2005). Postpartum females were brought to the infusion room in their home cage and left to habituate for 20 min. While pups remained in the home cage, mothers were infused with either OTR-A or saline and returned to their home cage containing their own litter which was then placed in an adjacent testing room. 20 min later, a weight matched (+/− 10 g) intruder female was introduced into the home cage (Neumann et al., 2001; Bosch et al., 2005). Each intruder was used for no more than two aggression tests, and none were used twice in the same day. Video recording began as soon as the intruder was placed in the cage and continued for 10 min. The following frequencies, latencies, and/or duration (when appropriate) of behaviors were assessed: intruder attacks (lunge, lunge plus wrestling bout and/or lunge plus pin), contact with intruder (included following or sniffing of the intruder), bites (on any part of the intruder’s body). One test session was discontinued because the intruder and some pups were wounded. Data from this animal were not included.

All behavioral tests were digitally recorded and videos were later scored blind by a trained observer using BEST Collection and BEST Analysis software (Education Consulting Inc., Hobe Sound, FL).

### Histology

**Figure 1:**
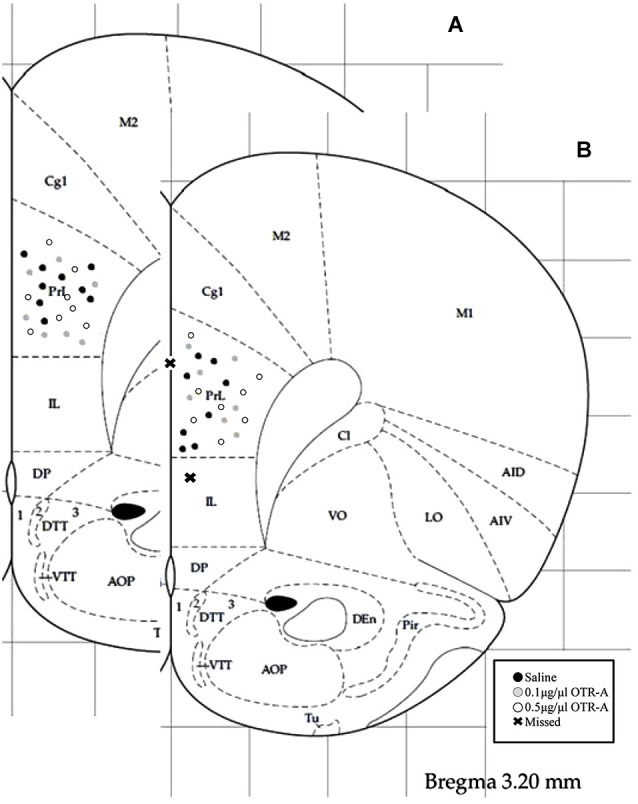
**Schematic representation of mPFC cannula placements**. Cannula tip placements were in the prelimbic region (PL) of the mPFC (AP: +3.2 mm, ML: ±0.5 mm, DV: −3.2 mm). Each dot indicates an individual subject. Infusions were bilateral but are represented unilaterally. Cannula placements for virgin **(A)** and postpartum **(B)** females receiving an infusion of saline, 0.1 μg/1 μl OTR-A or 0.5 μg/1 μl OTR-A. Animals with missed cannula placements in the infralimbic region (IL) or the ventricle were excluded from analyses. Adapted from Paxinos and Watson (1998).

Rats were overdosed with Euthasol and transcardially perfused with 4% paraformaldehyde 24–48 h after the completion of behavioral testing. Brains were removed, postfixed for 24 h and then sectioned on a Vibratome. 40-μm thick coronal sections were collected throughout the area of the cannula implant and stained with 0.2% cresyl violet for verification of correct placement (Figure 1). Those animals with cannula placements outside of the PL region of the mPFC (two postpartum females, one in the saline control group and one in the 0.1 μg/1 μl OTR-A group) were excluded from the study. Because these missed cannula placements included different drug and dosage groups, statistical analyses could not be completed in order to examine the behavioral effects of the OTR-A outside of the PL mPFC. However, because the PL cannula placements were not homogenous within this region, behavioral analysis of medial/lateral and dorsal/ventral PL cannula placements were performed for each experiment but revealed no differences. Thus, all cannula placements within a drug type or dose were grouped together regardless of location within the PL region. Examination under high magnification (100X) revealed limited to no damage at the tip of the cannula in any of the animals.

### Statistical analysis

All statistical analyses were performed using Graphpad Prism software version 5.01 (La Jolla, CA). Behavioral data in the maternal behavior and maternal aggression tests were analyzed separately in postpartum females using one-way analysis of variance (ANOVA). Anxiety-like behavior was analyzed using a 2 × 3 ANOVA with reproductive state (postpartum or virgin) and infusion type (saline, 0.1 μg/1 μl OTR-A, or 0.5 μg/1 μl OTR-A) as factors. Statistical significance for main effects and interactions were indicated by *p*-values < 0.05 and when significance was found were followed by Tukey’s HSD *post hoc* comparison test.

## Results

### Blocking OTR in the mPFC of postpartum rats alters maternal care

**Figure 2:**
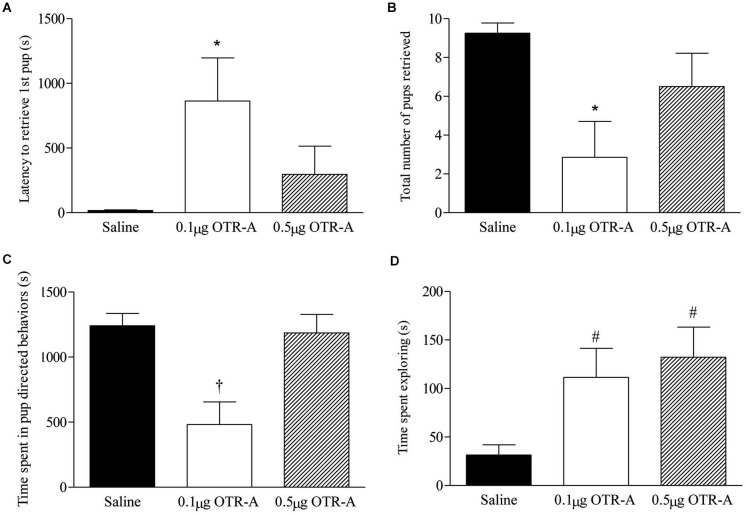
**Blocking OTR in the postpartum mPFC impairs maternal behavior.** Postpartum females receiving the lower 0.1 μg/μl dose of the OTR-A in the mPFC took longer to retrieve the first pup **(A)** and retrieved fewer pups **(B)** as compared to saline controls. The lower dose of the OTR-A in the mPFC also decreased the amount of time spent in pup directed behaviors **(C)**. Both the low and high dose of OTR-A increased home cage exploratory behavior **(D)**. Bars represent mean ± SEM; * *P* < 0.05 0.1 μg/μl OTR-A vs. saline, † *P* < 0.05 0.1 μg/μl OTR-A vs. saline and 0.5 μg/μl OTR-A, # *P* < 0.05 0.1 μg/μl OTR-A and 0.5 μg/μl OTR-A vs. saline.

Blocking OTR in the postpartum PL mPFC impaired pup retrieval behavior by increasing the latency to retrieve the first pup (*F*_2,20_ = 3.74, *p* < 0.05; Figure 2A) and decreasing the number of pups retrieved (*F*_2,20_ = 4.71, *p* < 0.05; Figure 2B). *Post hoc* analysis revealed that postpartum females infused with the lower 0.1 μg/μl dose of the OTR-A took longer to retrieve their first pup and retrieved fewer pups as compared to saline controls (*p*’s < 0.05). Pup directed behaviors were also impaired by blocking OTR in the PL mPFC of postpartum females (*F*_2,20_ = 8.98, *p* < 0.05; Figure 2C) with *post hoc* analysis showing that those infused with the lower dose of the OTR-A spent less time engaged in pup-directed behaviors as compared to those infused with saline or the higher OTR-A dose (*p*’s < 0.05). In addition to affecting maternal behavior, OTR antagonism also altered exploratory behavior (*F*_2,20_ = 4.60, *p* < 0.05; Figure 2D) such that both the lower and higher dose of the OTR-A increased the amount of time spent exploring the home cage when compared to saline controls (*p*’s < 0.05). Rearing and self-grooming were not affected by either dose of the OTR-A (*p*’s > 0.05; data not shown).

### Blocking OTR in the mPFC of postpartum rats increases maternal aggression

**Figure 3:**
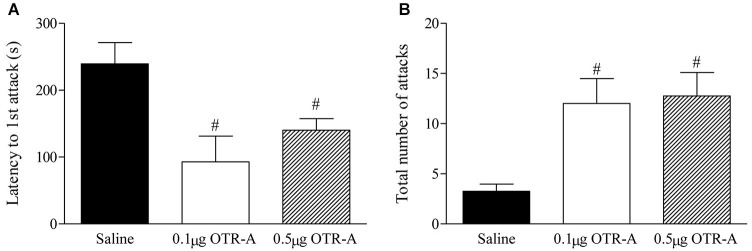
**Blocking OTR in the postpartum mPFC enhances maternal aggression.** Postpartum females infused with either the lower 0.1 μg/μl or higher 0.5 μg/μl dose of the OTR-A in the mPFC displayed a significant decrease in latency to attack the intruder **(A)** and a greater number of total intruder attacks **(B)** as compared to saline controls. Bars represent mean ± SEM; # *P* < 0.05 0.1 μg/μl OTR-A and 0.5 μg/μl OTR-A vs. saline.

Blocking OTR in the postpartum PL mPFC enhanced maternal aggression as revealed by a decrease in the latency to attack the intruder (*F*_2,20_ = 6.29, *p* < 0.05; Figure 3A) and increased number of intruder attacks (*F*_2,20_ = 7.38, *p* < 0.05; Figure 3B). *Post hoc* analysis showed that postpartum females infused with either the lower 0.1 μg/μl or higher 0.5 μg/μl dose of the OTR-A took less time to attack the intruder (*p*’s < 0.05; Figure 3A) and attacked the intruder more (*p*’s < 0.05; Figure 3B) as compared to saline controls. There was also a trend for the higher dose of the OTR-A to increase the time spent in contact with the intruder (*p* = 0.07; data not shown) but the number of intruder bites was not significant (*p* > 0.05; data not shown).

### Attenuated anxiety during the postpartum period is prevented by blocking OTR in the mPFC

**Figure 4:**
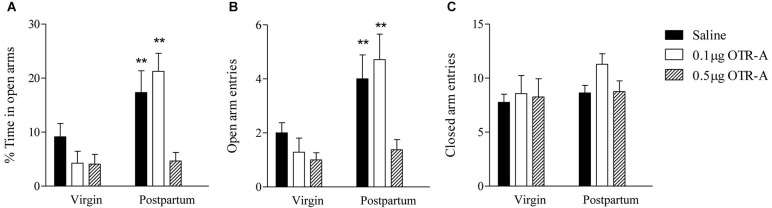
**Blocking OTR in the mPFC enhances postpartum anxiety, but has no effect on anxiety in virgin females.** Postpartum females infused with saline or the lower 0.1 μg/μl dose of the OTR-A in the mPFC spent a greater percentage of time in the open arms and **(A)** made more open arm entries **(B)** as compared to virgins. In contrast, postpartum females receiving the higher 0.5 μg/μl dose of the OTR-A displayed a decrease in the percentage of time spent in the open arms **(A)** and made fewer open arm entries **(B)** as compared to postpartum females infused with saline or low dose OTR-A. Locomotor activity, as measured by the number of closed arm entries **(C)**, was not altered. None of the virgin groups differed significantly from one another **(A, B, C)**. Bars represent mean ± SEM; ** *P* < 0.01 postpartum saline and postpartum 0.1 μg/μl OTR-A vs. all other groups.

Reproductive state and OTR-A infusion into the PL mPFC had significant effects on anxiety-like behavior in the EPM. For the percentage of time spent in the open arms of the EPM (Figure 4A), there was a main effect of reproductive state such that postpartum females spent more time in the open arms than virgins (*F*_1,40_ = 15.07, *p* < 0.0005) indicating lower anxiety in postpartum females. There was also a significant main effect of infusion type (*F*_2,40_ = 6.97, *p* < 0.005) and a significant reproductive state by infusion type interaction (*F*_2,40_ = 4.5, *p* < 0.05). *Post hoc* analysis revealed that in postpartum rats, the group receiving the higher 0.5 μg/μl dose of the OTR-A spent less time in the open arms as compared to both the saline and 0.1 μg/μl OTR-A groups (*p*’s < 0.01), which did not differ. Postpartum females given the higher 0.5 μg/μl dose of the OTR-A did not differ from virgins in the percentage of time spent in the open arms (*p*’s > 0.05). None of the virgin groups significantly differed from each other.

The number of entries made into the open arms of the EPM (Figure 4B) also showed a significant main effect of reproductive state such that postpartum females made more open arm entries than virgins again indicating reduced anxiety in postpartum females (*F*_1,40_ = 15.21, *p* < 0.0005). There was also a significant main effect of infusion type (*F*_2,40_ = 6.08, *p* < 0.005) and a marginally significant reproductive state by infusion type interaction (*F*_2,40_ = 3.11, *p* = 0.06). *Post hoc* analysis revealed that in postpartum rats, the group receiving the higher 0.5 μg/μl dose of the OTR-A made fewer open arm entries as compared to both the saline and the lower 0.1 μg/μl OTR-A groups (*p*’s < 0.01), which did not differ. Postpartum females given the higher 0.5 μg/μl dose of the OTR-A did not differ from virgins in the number of open arm entries (*p*’s > 0.05). None of the virgin groups significantly differed from each other.

For the number of closed arm entries in the EPM (Figure 4C), there was no main effect of reproductive state (*F*_1,40_ = 1.96, *p* > 0.05) or infusion type (*F*_2,40_ = 1.17, *p* > 0.05) and no significant reproductive state by infusion type interaction (*F*_2,40_ = 0.47, *p* > 0.05) indicating that locomotor activity was unaltered. In contrast to the EPM, there were no significant main effects or interactions for any behaviors measured in the open field (*p*’s > 0.05; data not shown).

## Discussion

The present work shows that OTR activity within the PL region of the mPFC modulates maternal care, maternal aggression, and anxiety-like behavior during the early/mid postpartum period. We observed that infusion of a highly specific OTR-A into the mPFC of postpartum females impaired pup retrieval and reduced the display of pup-directed behaviors. Blockade of OTR within the mPFC was also sufficient to increase maternal aggressive behavior and to prevent the reduction in anxiety typically observed during the postpartum period. Together, these findings identify the mPFC as a common brain site for the regulation of numerous postpartum-related behaviors by OT.

### mPFC OT and maternal care

OT has long been examined for its role in regulating maternal care behaviors. In general, OT has been shown to be critical for the induction and maintenance of maternal behavior (Pedersen and Prange, 1979; van Leengoed et al., 1987; Insel, 1990; Pedersen et al., 1994, 2006; Shahrokh et al., 2010; Bosch and Neumann, 2012). There are numerous brain sites where OT acts to influence maternal care including the MPOA (Pedersen et al., 1994; Bosch and Neumann, 2012), VTA (Pedersen et al., 1994; Shahrokh et al., 2010), and OB (Yu et al., 1996). The mPFC is interconnected with each of these regions (Vertes, 2004; Peters et al., 2009; Numan and Woodside, 2010) and has itself been shown to regulate some aspects of maternal behavior (Afonso et al., 2007; Febo et al., 2010). Thus, the present findings extend this prior work and identify the mPFC as another component of the “maternal circuit” (Numan and Woodside, 2010) where OT activity can influence maternal care. Specifically, mothers infused with the lower, but not higher, dose of the OTR-A in the mPFC exhibited impairments in pup retrieval and other pup directed behaviors which included pup licking, pup contact, and nursing. It is worth noting that both doses of the OTR-A increased home-cage exploration although the total time spent doing so was very low and thus unlikely to account for differences in maternal care behaviors (Afonso et al., 2007; Curley et al., 2012). Instead, OT in the mPFC may modulate motivation or other functions of the mPFC such as attention that may be important for the proper display of pup directed maternal care (Afonso et al., 2007; Pereira and Morrell, 2011).

### mPFC OT and maternal aggression

Previous studies have linked maternal aggression to activation of the mPFC (Gammie et al., 2004; Nephew et al., 2009) and thus implicate the mPFC in the modulation of aggressive behavior during the postpartum period. Here we show that maternal aggression was affected when OTR activity in the postpartum mPFC was blocked. In particular, postpartum females treated with either dose of the OTR-A displayed a reduction in the latency to attack the intruder and an increased number of intruder attacks. However, it is worth considering that the ability to detect an increase in maternal aggression following OTR-A administration may be related to the relatively low levels of aggression in saline treated mothers which could in turn be due to the timepoint examined or the use of a female intruder. Thus, our results tentatively suggest an inhibitory effect of OT on the aggressive behavior of postpartum females and implicate the mPFC, in addition to the PVN (Giovenardi et al., 1998; Bosch et al., 2004), BNST (Consiglio et al., 2005) and CEA (Lubin et al., 2003; Bosch et al., 2005; Consiglio et al., 2005), as a brain site for OT’s actions on maternal aggression.

The relationship between OT and maternal aggression is controversial. Because the onset of maternal aggression coincides with high levels of OT, it is reasonable to predict that OT would increase maternal aggressive behavior. Consistent with this are studies demonstrating a reduction in various components of maternal aggression following electrolytic lesions of the PVN (Consiglio and Lucion, 1996) but an increase following elevation of OT in the PVN (Bosch et al., 2005). However, other work contradicts these findings and instead shows that potentiated aggressive behavior during the postpartum period correlates with *decreased* OT activity. For example, excitotoxic lesions of the PVN or local inhibiton of OT synthesis increases the biting frequency of postpartum females against a male intruder (Giovenardi et al., 1998). Similarly, infusion of an OTR-A into the CeA of postpartum rats increases attack frequency (Lubin et al., 2003). An inverse relationship between OT and maternal aggression has also been observed following OT infusion into the amygdala which was shown to decrease maternal aggressive behavior (Consiglio et al., 2005; Caughey et al., 2011). Indeed, maternal aggression has been inversely correlated to OT levels in the amygdala (Johns et al., 1994, 1998; Lubin et al., 2003; Bosch et al., 2005). These discrepancies have been attributed to the use of different rat strains and differences in the experimental design (i.e., timing of testing relative to administration of the OTR-A or relative to birth, male vs. female intruder) (Bosch and Neumann, 2012) but further studies are needed.

Although it may seem contradictory that OT acting in the mPFC facilitates maternal care yet inhibits maternal aggression, our results are consistent with the importance of OT in promoting pro-social behaviors (Insel and Young, 2001; Porges, 2003; Macdonald, 2012). In this regard, it is important to consider that while maternal care is a relationship between the mother and the pups, maternal aggression is an activity directed towards an adult intruder (Giovenardi et al., 1998). Moreover, while maternal care implies affiliation and pair bonding, aggressive behaviors by definition do not. Therefore, the nature of the two behaviors is different and involves different neural circuitries (Lonstein and Gammie, 2002; Gammie, 2005; Bosch and Neumann, 2012), and thus it seems plausible that maternal care and maternal aggression may be differentially sensitive to OTR blockade in the mPFC.

### mPFC OT and anxiety-like behaviors

The postpartum period has repeatedly been shown to be a time that is associated with reduced anxiety (Fleming and Luebke, 1981; Hard and Hansen, 1985; Neumann et al., 2000a; Lonstein, 2005a, 2007; Figueira et al., 2008; Macbeth and Luine, 2010; Jurek et al., 2012). We again support these findings here by showing that postpartum rats spend a greater percentage of time in the open arms of an EPM and make more entries into the open arms as compared to diestrus virgins. OT has frequently been examined for its role in modulating anxiety (McCarthy et al., 1996; Windle et al., 1997; Neumann et al., 2000b; Bale et al., 2001; Waldherr and Neumann, 2007; Neumann and Landgraf, 2012) and a recent study has found that OT in the mPFC of virgin male and female rats is anxiolytic (Sabihi et al., 2014). However, investigation into specific sites in the brain where OT acts to reduce anxiety-like behaviors in postpartum females has been limited. The PAG (Figueira et al., 2008), PVN (Jurek et al., 2012), and amygdala (Bosch et al., 2005) are the only regions that have been implicated in the OT-mediated regulation of anxiety in postpartum females. All of these regions are interconnected with the mPFC (Vertes, 2004; Peters et al., 2009; Numan and Woodside, 2010) and thus our findings suggest that the mPFC may be part of a complex network that modulates postpartum anxiety-like behavior.

In contrast to postpartum females, OTR-A infused into the mPFC of diestrus virgins did not affect the percentage of time or number of entries into the open arms of the EPM. These results are in line with previous findings demonstrating that OTR blockade decreases the percentage of time spent in open-arms by postpartum, but not virgin female rats (Neumann et al., 2000a,b; Figueira et al., 2008; Sabihi et al., 2014). The differential effects of OTR antagonism in postpartum vs. virgin females likely reflects reproductive differences in OT release and OTR expression in many brain regions (Bosch and Neumann, 2012). While postpartum females exhibit elevated OTR expression and/or peptide release, virgin females do not (Insel, 1990; Landgraf et al., 1992; Neumann et al., 1993; Pedersen et al., 1994; Francis et al., 2000; Bosch et al., 2010; Caughey et al., 2011). Therefore, perhaps it is not so surprising that blocking OTR within the mPFC only impacted anxiety-like behavior in postpartum females. It is important to point out however that anxiety-like behavior was tested in postpartum females on PD5 after a second infusion of the OTR-A whereas virgin females were tested after a single infusion of the OTR-A and these differences could also have contributed to the effects observed.

In the OF, the anxiogenic actions of the OTR-A were undetectable in both postpartum and virgin females. Although the EPM and OF both have an exploratory component, the EPM is considered a more sensitive test of anxiety (Hilakivi and Lister, 1990) and behavior in one test does not always predict behavior in the other (Bale et al., 2001; Bhatnagar et al., 2004; Sabihi et al., 2014). It is also possible that the inconsistencies in the OF may be related to variations in the testing conditions known to influence OF behavior (Lapiz-Bluhm et al., 2008) or differential sensitivity of the OF to the OTR-A which may require different doses than those used here for an anxiogenic effect to be revealed.

### Differential sensitivity of maternal behavior, maternal aggression, and postpartum anxiety-like behavior to mPFC OTR blockade

The effects of the OTR-A on the various postpartum behaviors measured here were largely dose specific. The effects of OTR blockade on maternal care occurred when a 0.1 μg/μl dose, but not a 0.5 μg/μl dose, of the OTR-A was used. The ability of the lower, but not higher, dose of the OTR-A to modify maternal behavior is consistent with the dose-dependent but often nonlinear effect found for increased doses of neuropeptides or their antagonists (Figueira et al., 2008; Leuner et al., 2012). Such nonlinear effects may be due to many factors including a refractory state of receptors at higher doses (Landgraf and Neumann, 2004). However, this seems unlikely as the higher dose of the OTR-A seemed to have more widespread effects on maternal aggression and exclusively impacted anxiety-like behavior. Another possible explanation for the dose-dependent effects of the OTR-A may be related to the above mentioned fact that maternal care is considered an affiliative behavior whereas anxiety and aggression are fear/defensive behaviors and thus may be differentially regulated by OT in the mPFC. Finally, like OT, the closely related neuropeptide vasopressin has also been implicated in the initiation and maintenance of maternal care behaviors (Bosch and Neumann, 2008, 2012; Bosch et al., 2010). Thus, even though OTR are blocked with the OTR-A, vasopressin’s actions may allow for complex compensatory effects that could be behavior specific.

In addition to dose specificity, the possibility of subregional specificity must also be considered. The mPFC of the rodent brain consists of three subregions—the infralimbic (IL) cortex, PL cortex, and anterior cingulate cortex (Cg1; Heidbreder and Groenewegen, 2003). Here we targeted only the PL mPFC because it has been most consistently linked to maternal care and aggression (Nephew et al., 2009; Febo et al., 2010; Pereira and Morrell, 2011; Febo, 2012) as well as OT regulation of social (Young et al., 2014) and anxiety behaviors (Sabihi et al., 2014). However, the various subregions of the mPFC show different patterns of connectivity with subcortical and cortical structures which could lead to different behavioral outcomes following OTR blockade. Given the injected volume and expected diffusion of the OTR-A, OTR blockade was likely limited to the PL mPFC. The PL region extends approximately 1500 μm mediolaterally, 2000–2500 μm dorsoventrally, and 2700–3000 μm rostrocaudally. OT and OTR-A are structurally similar to vasopressin, which diffuses in an estimated sphere of 1000 μm from the tip of the injector cannula per 1 μl microinfusion (Kovács et al., 1980, 1982). Given its smaller molecular mass (Manning et al., 2012), the OTR-A presumably spread from all directions of the injector cannula an equal or slightly greater distance than an identical volume of vasopressin. Together with the anatomical accuracy of cannula tip placement, we believe that the OTR-A diffused into the majority of the PL region without diffusing into neighboring structures. Nonetheless, without experimentally measuring diffusion of the OTR-A or the behavioral outcomes of blocking OTR in other regions of the mPFC, we cannot eliminate the possibility that leakage to nearby structures may mediate some of the observed effects.

### Interactions among maternal care, maternal aggression, and anxiety

The interactions among anxiety, maternal care, and maternal aggression are complex. It is commonly assumed that attenuated anxiety may be required for the adequate display of maternal care (Lonstein, 2007). Consistent with this, higher maternal anxiety has been linked to aberrant maternal care in humans (Turner et al., 2003) as well as in non-human primates who exhibit impaired infant retrieval, increased maternal rejection, and abuse of infants (Troisi and D’Amato, 1994; Saltzman and Maestripieri, 2011) Similarly, a relationship between increased anxiety-like behavior and impaired maternal care has been demonstrated in postpartum rats (Fleming and Luebke, 1981; Boccia and Pedersen, 2001; Bosch et al., 2005; Lonstein, 2005a). However, there are discrepancies in the literature (Lonstein, 2007). For example, amongst postpartum rodents selected for higher anxiety-like behavior there appears to be elevated levels of some maternal behaviors (Bosch, 2011). In the present study, control mothers receiving a saline infusion in the mPFC displayed high levels of maternal care and low anxiety but this relationship did not hold true following OTR-A administration. Indeed, lower levels of maternal care and anxiety were observed in postpartum rats given the low dose of the OTR-A whereas the high dose of the OTR-A led to increased anxiety without affecting maternal care. Thus, our results show no direct link between anxiety-like behavior and maternal behavior. Minimally these data, along with others (Curley et al., 2012), highlight the challenge of establishing the nature and direction of the anxiety-maternal behavior relationship.

Like maternal care, it has been suggested that heightened aggression may too depend on a reduction in anxiety such that a less anxious rat will be less hesitant to attack a potentially threatening and normally fear-evoking stimulus (Lonstein et al., 1998; Caughey et al., 2011). Although the hypothesis that heightened aggression during the postpartum period requires a concomitant reduction in fear and anxiety is logical, there are numerous examples where such a simple association does not exist (Boccia and Pedersen, 2001; Bosch et al., 2005; Lonstein, 2005b) including the present results showing higher levels of aggression along with low and high levels of anxiety following administration of the OTR-A at low and high doses, respectively. Thus, even though reduced anxiety is often found in postpartum rodents, it seems that in many cases it is neither sufficient nor necessary for their heightened maternal responsiveness or aggression (Lonstein, 2005b, 2007).

## Concluding remarks

A large body of work over many years has identified an extensive network of brain sites underlying the behavioral effects of OT during the postpartum period (Lonstein and Gammie, 2002; Gammie, 2005; Numan and Woodside, 2010; Bosch and Neumann, 2012). While the brain regions on which OT acts to regulate maternal care, maternal aggression, and postpartum anxiety are not necessarily identical, some overlaps exist (Gammie, 2005; Bosch and Neumann, 2012). Our results may be the first to reveal that OT in the maternal mPFC modulates maternal care, maternal aggression, and postpartum anxiety, although the relationship among these behaviors is complicated and further investigation examining OT release and OTR expression is required to refine our understanding of OT’s actions in the postpartum mPFC. Nonetheless, these data provide new insights into the neural circuitry of OT-mediated postpartum behaviors.

## Authors and contributors

Sara Sabihi contributed to the study design and manuscript preparation in addition to performing experiments and data analysis. Benedetta Leuner contributed to the study design and manuscript preparation. Nicole E. Durosko and Shirley M. Dong performed the experiments and data analysis.

## Conflict of interest statement

The authors declare that the research was conducted in the absence of any commercial or financial relationships that could be construed as a potential conflict of interest.
